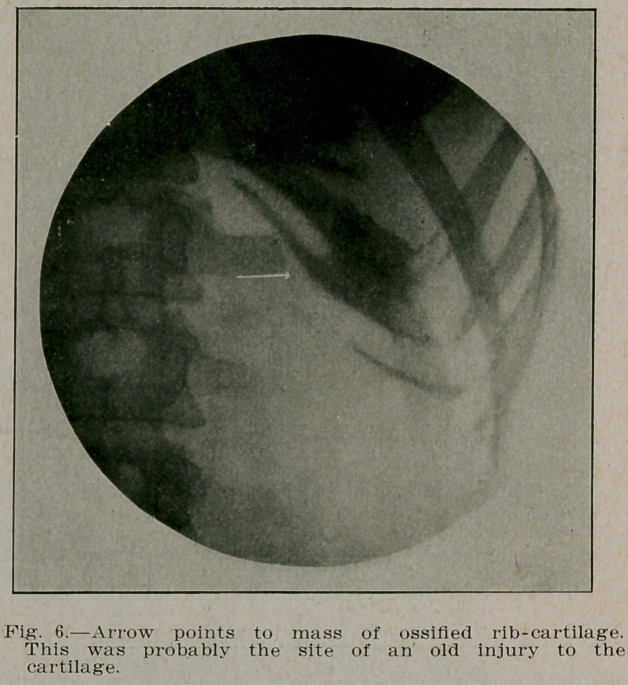# The Demonstration of Gall-Stones

**Published:** 1914-08

**Authors:** 


					﻿The Demonstration of Gall-Stones by the Roentgen Ray, by
A. W. George, M. I)., and I. Gerber, M. D., Boston. “Boston
Medical and Surgical Journal,’’ April 30, 1914. Up to within
the past year the demonstration of gall-stones upon plates by
the Roentgen method has been regarded generally, as a rare
curiosity. Of late, however, this problem has reached a posi-
tion, at least in this country, so that the authors feel that the
Roentgen method should be used more generally as a depend-
able aid to the diagnosis of gall-stones. It is advisable to ex-
amine the gall-bladder region for stones prior to every bis-
muth examination of the alimentary tract.
Stones are frequently found where their presence was not
even suspected, and where the entire trouble was attributed to
some other organ, such as the stomach.
The demonstration of the stones depends upon the amount
of calcium present. The more calcium there is, the easier it is
to show the shadow of the stones. More recently the authors
have succeeded in demonstrating stones that are composed
largely or almost entirely of cholesterin. These stones are ex-
tremely difficult to show; and their detection depends upon
care in the examination of the right upper quadrant, ajid a
larger number of plates taken under the most favorable condi-
tions. It is these stones which have been generally missed
because of lack of care in looking for them. The authors be-
lieve that with proper care and enough plates gall-stones can
be demonstrated by the Roentgen method in nearly every case
of gall-bladder disease of long standing where stones are really
present.
The chief sources of error in the interpretation of the plates
are renal calculi, calcified mesenteric glands, and costo-
chondral ossification. These can be differentiated by proper
technique.
				

## Figures and Tables

**Fig. 1. f1:**
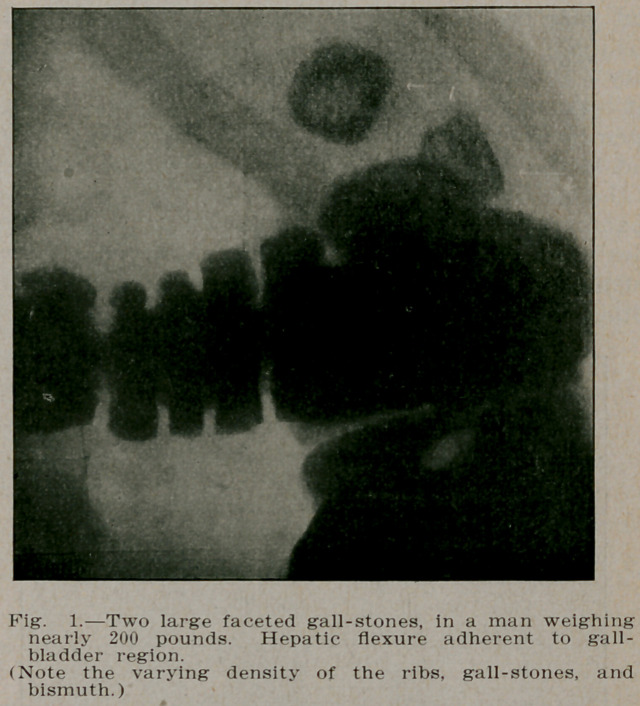


**Fig. 3. f2:**
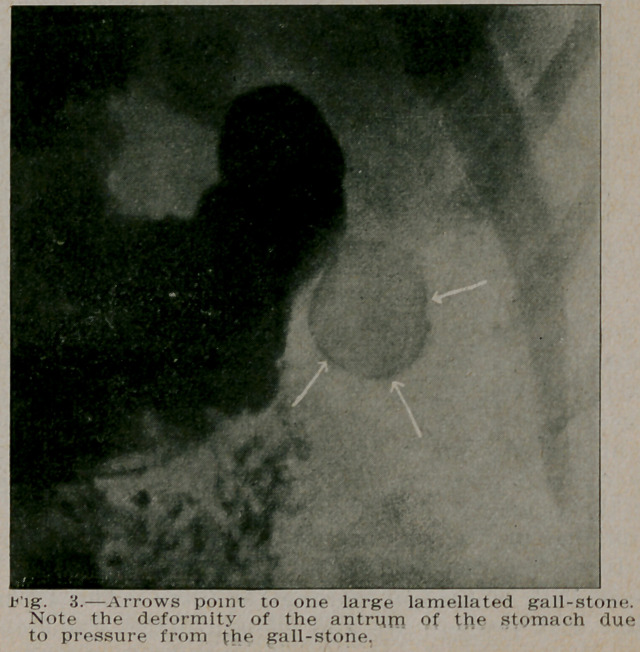


**Fig. 2. f3:**
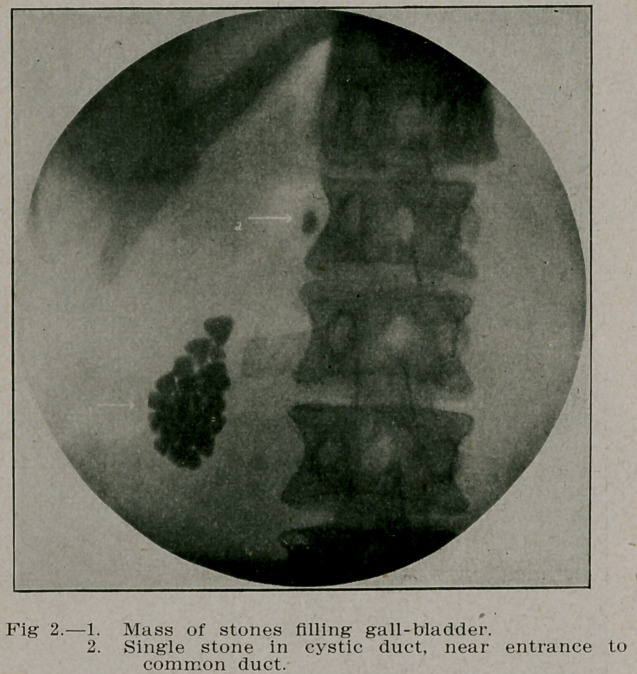


**Fig. 4. f4:**
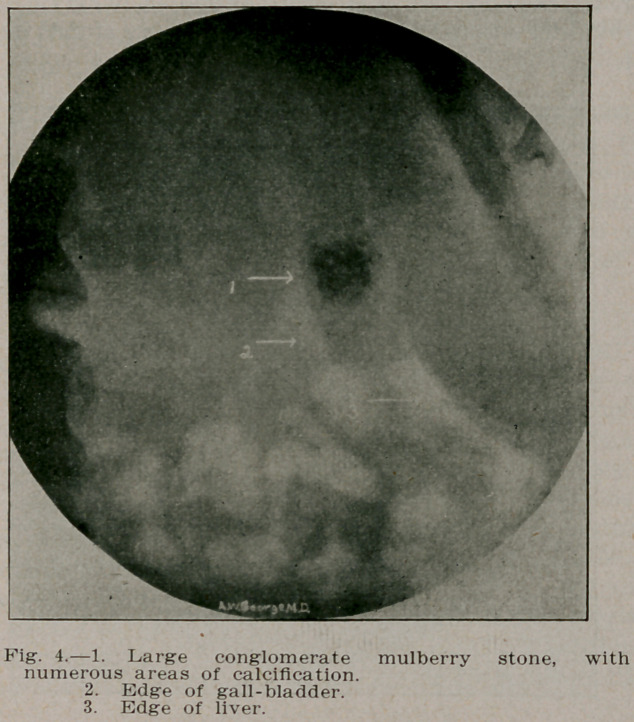


**Fig. 5. f5:**
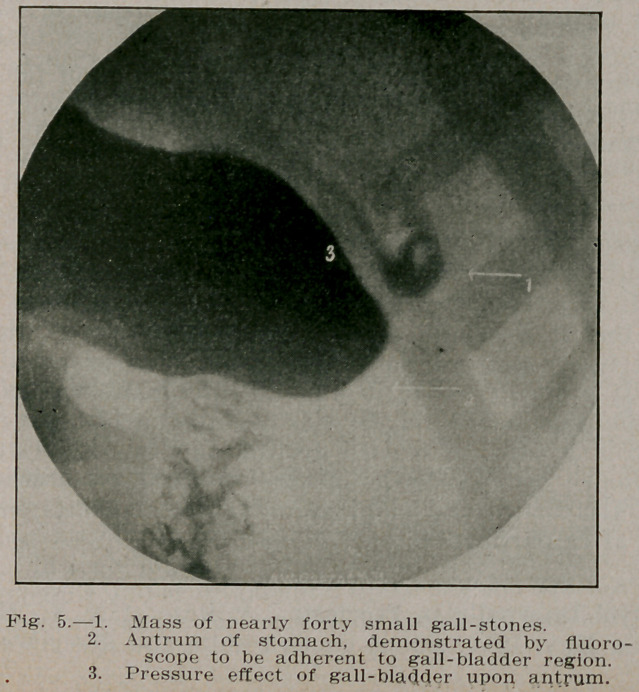


**Fig. 6. f6:**